# Discover cervical disc arthroplasty versus anterior cervical discectomy and fusion in symptomatic cervical disc diseases: A meta-analysis

**DOI:** 10.1371/journal.pone.0174822

**Published:** 2017-03-30

**Authors:** Lei Shangguan, Guang-Zhi Ning, Yu Tang, Zhe Wang, Zhuo-Jing Luo, Yue Zhou

**Affiliations:** 1 Department of Orthopaedics, Xinqiao Hospital, The Third Military Medical University, Shapingba District, Chongqing, China; 2 Department of Orthopaedics, Xijing Hospital, The Fourth Military Medical University, Xi'an, China; 3 Department of Orthopaedics, Tianjin Medical University General Hospital, Heping District, Tianjin, China; Universita degli Studi di Palermo, ITALY

## Abstract

**Objective:**

Symptomatic cervical disc disease (SCDD) is a common degenerative disease, and Discover artificial cervical disc, a new-generation nonconstrained artificial disk, has been developed and performed gradually to treat it. We performed this meta-analysis to compare the efficacy and safety between Discover cervical disc arthroplasty (DCDA) and anterior cervical discectomy and fusion (ACDF) for SCDD.

**Methods:**

An exhaustive literature search of PubMed, EMBASE, and the Cochrane Library was conducted to identify randomized controlled trials that compared DCDA with ACDF for patients suffering SCDD. A random-effect model was used. Results were reported as standardized mean difference or risk ratio with 95% confidence interval.

**Results:**

Of 33 articles identified, six studies were included. Compared with ACDF, DCDA demonstrated shorter operation time (P < 0.0001), and better range of motion (ROM) at the operative level (P < 0.00001). But no significant differences were observed in blood loss, neck disability index (NDI) scores, neck and arm pain scores, Japanese orthopaedic association (JOA) scores, secondary surgery procedures and adverse events (P > 0.05). Subgroup analyses did not demonstrated significant differences.

**Conclusion:**

In conclusion, DCDA presented shorter operation time, and better ROM at the operative level. However, no significant differences were observed in blood loss, NDI scores, neck and arm pain scores, JOA scores, secondary surgery procedures and adverse events between the two groups. Additionally, more studies of high quality with mid- to long-term follow-up are required in future.

## Introduction

Symptomatic cervical disc disease (SCDD) is a common disease around the world. According to a previous study[1], the hospitalization rates of surgical procedures for SCDD increased from an estimated 41000 to 76000 every year with a 85% increase during 1980 to 1990. In fact, there were 50–60 patients who chose surgery treatment for SCDD per 10,000 inhabitants every year[2]. Anterior cervical discectomy and fusion (ACDF) has been considered as a “gold standard” of surgical procedure for the treatment of SCDD[3]. Lied et al[3] declared in their study that ACDF was effective in alleviating radicular pain for patients with SCDD. However, this surgical procedure has been hampered by a great amount of complications, such as hypermobility, pseudoarthrosis, dysphagia, and adjacent segment degeneration[4]. For this reason, cervical disc arthroplasty (CDA), a new procedure, was developed and performed gradually[5–9]. Additionally, evidences from many studies have shown that CDA has several theoretical advantages in maintaining disc height and keeping the physiological motion[10–12].

Many kinds of artificial cervical disc have been produced and used for CDA. Discover artificial cervical disc (DePuy Spine, Raynham, Massachusetts), a new-generation nonconstrained artificial disc with a built in 7.0° lordotic angle design, is expected to allow restoration of cervical sagittal alignment[13]. And up to now, it has been used gradually for the treatment of SCDD. However, Thaler et al[14] demonstrated that only about 40% of Discover footprints could match anatomic dimensions, such as anteroposterior and mediolateral diameters, and dissipate the axial load regularly. Due to this, the vertebral displacement may occur after Discover cervical disc arthroplasty (DCDA). Conversely, Luo et al[15] showed that DCDA was superior to ACDF in the mid-term effect. Therefore, the efficacy and safety of DCDA was still controversial.

Although lots of meta-analyses have been conducted to compare CDA with ACDF for the treatment of SCDD, the CDA groups comprised diverse types of cervical disc prostheses, which may influence the contrastive results between the two groups, and no review comparing DCDA with ACDF was found. Considering the special characteristics of DCDA, we performed this meta-analysis to compare the efficacy and safety between DCDA and ACDF for treating SCDD and provide reliable evidence for the clinicians.

## Materials and methods

### Search strategy and study selection

We conducted an exhaustive literature search of PubMed, EMBASE, and the Cochrane Library to identify randomized controlled trials that compared DCDA with ACDF for patients suffering SCDD. Mesh terms and text words were combined in the literature retrieval. The search terms concerning SCDD were combined with the terms regarding both DCDA and ACDF. The detailed search information was showed in S1 Table. We did not limit the languages or publication date. References of the relevant studies were also reviewed for additional worthy literatures. The literature search was last updated on August 20, 2016. Based on the titles and abstracts, two investigators picked out the potential eligible studies. And then the full text of the remaining studies were reviewed for eligibility. Any divergence was resolved through consensus.

### Eligibility criteria

(1) Participants: The study population consisted of patients who were adult, had SCDD (including radiculopathy, myelopathy, or disc herniation), and were unresponsive to nonoperative treatment for at least 6 weeks or longer.

(2) Interventions: The intervention in the experimental group was CDA with the prostheses of Discover. Other types of prostheses were excluded.

(3) Comparisons: The intervention in the control group was ACDF.

(4) Outcomes: Studies were qualified when at least one of the following outcomes were given: operation time, blood loss, neck disability index (NDI) scores, neck and arm pain scores, range of motion (ROM) at the operative level, Japanese orthopaedic association (JOA) scores, secondary surgical procedures, and adverse events.

(5) Study design: Only randomized controlled trials were regarded as eligible in the present study. Multiple publications of the same trial were excluded.

### Data extraction and outcome measures

Two reviewers independently performed the data extraction from the qualified studies. Any disagreement was resolved by discussion or by consulting a third reviewer. The indispensable study characteristics involving details of methodology, sample size, age, sex distribution, experimental and control interventions, and outcomes were extracted.

The outcome measures of interest consisted of surgical parameters (operation time and blood loss), and clinical indexes (NDI scores, neck and arm pain scores, ROM at the operative level, JOA scores, secondary surgical procedures, and adverse events).

### Risk of bias assessment

Two reviewers applied the risk of bias tool to appraise all the included literatures according to the Cochrane Handbook for Systematic Reviews of Interventions (version 5.1.0), respectively. The parameters of appraisal covered random sequence generation, allocation concealment, blinding of participants and personnel, blinding of result assessor, incomplete result data, selective result reporting and other bias (baseline balance). All of the domains were defined as low risk of bias, high risk of bias, or unclear risk of bias.

### Quality of evidence assessment

The strength of evidence for each pooled outcome was rated according to the Grades of Recommendation, Assessment, Development, and Evaluation (GRADE) approach. On the basis of the assessment of five major criteria: risk of bias, inconsistency, indirectness, imprecision and publication bias, the quality of outcomes was rated as high, moderate, low, or very low quality. GRADE Pro version 3.6 was used to generate summary tables.

### Statistical analysis

In this meta-analysis, we calculated the risk ratio (RR) and its 95% confidence interval (CI) for the dichotomous data and the standardized mean difference (SMD) and its 95% CI for the continuous data. A random-effect model was used for the present meta-analysis[16]. The I^2^ statistic[17] was used to assess the heterogeneity across studies. It indicated significant heterogeneity when an I^2^ value surpassed 50%. Based on duration of follow-up (short-term (1–3 years), mid-term (4–5 years)) and target level (single-level or two-level or mixed-level), subgroup analyses were performed for the clinical indexes. We performed the sensitivity analyses using a fixed-effect model, and excluding the largest and most weighted trials. Furthermore, we conducted meta-regression analyses to appraise the potential effect of mean age and gender on the clinical indexes. The Egger’s linear regression test and funnel plots were used to examine the possibility of publication bias when more than ten studies were included[18]. P < 0.05 signified statistically significant differences. The statistical analyses was performed by Review Manager version 5.3 (The Nordic Cochrane Centre, The Cochrane Collaboration, Copenhagen, 2014) and Stata version 12.0 (Stata Corp, College Station, TX).

## Results

### Study search

**Fig 1:**
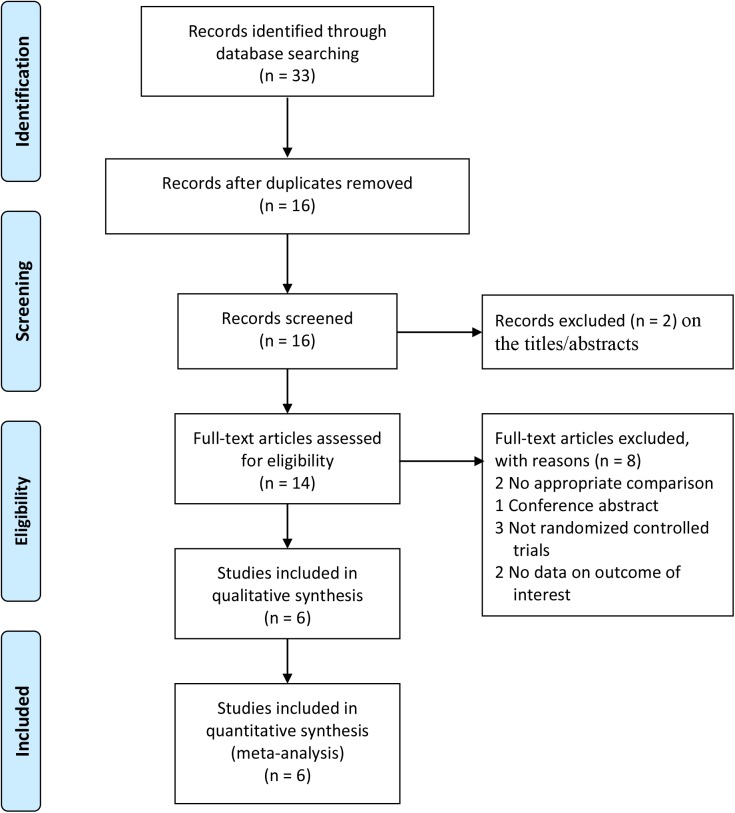
Flow diagram of study selection.

Fig 1 shows a summary of the study selection process. There were altogether 33 relevant literatures inspected from the electronic search. 17 studies were excluded because they were duplicates. After assessing the titles and abstracts, two studies were eliminated because they did not meet the eligibility criteria. After verifying the full-text of the remaining 14 studies, six randomized controlled trials[13, 15, 19–22] with 505 patients finally were included in this meta-analysis. Furthermore, we also classified the duration of follow-up as short-term (1–3 years) or mid-term (4–5 years).

### Study characteristics

Table 1 summarizes the main characteristics of studies included. The baseline information of two groups were balanced and comparable. Of the six identified randomized controlled trials, one study[22] was a multicenter trial and the remaining trials were all from single trial site. Among the six studies, four studies were conducted in China[13, 15, 19, 21], one study was conducted in Sweden[22] and the other one was from Croatia[20]. Furthermore, the duration of follow-up were less than 48 months in five studies[13, 19–22] and one study[15] reported the outcomes at the end of 48 months.

**Table 1:** Baseline characteristics of studies included in the meta-analysis.

Source	Study characteristics	Number of patients		Age (Mean±SD) (years)		Sex(male/female)		Number of cervical levels	Follow up (months)	Number of trial sites
		DCDA	ACDF	DCDA	ACDF	DCDA	ACDF			
Chen 2013	November 2008 to October 2010	16	16	43.2±10.2	46.5±7.9	9/7	8/8	Single-level	24	1
Luo 2015	January 2009 to October 2011	34	37	47.2±6.5	46.3±7.1	18/16	20/17	Single-level	48	1
Rozankovic 2016	October 2008 to June 2010	51	50	41.32±8.8	41.94±9.36	25/26	25/25	Single-level	24	1
Shi 2016	September 2009 to December 2012	60	68	46.5±6.8	47.4±7.0	36/35	24/33	Single-level	24	1
Skeppholm 2015	April 2007 to May 2010	81	70	46.7±6.7	47.0±6.9	40/41	33/37	Mixed-level	24	2
Sun 2016	December 2009 to December 2011	14	16	46.79±5.15	48.13±5.98	9/5	11/6	Two-level	32.4	1

DCDA: Discover cervical disc arthroplasty, ACDF: anterior cervical discectomy and fusion.

### Risk of bias in the included studies

**Fig 2:**
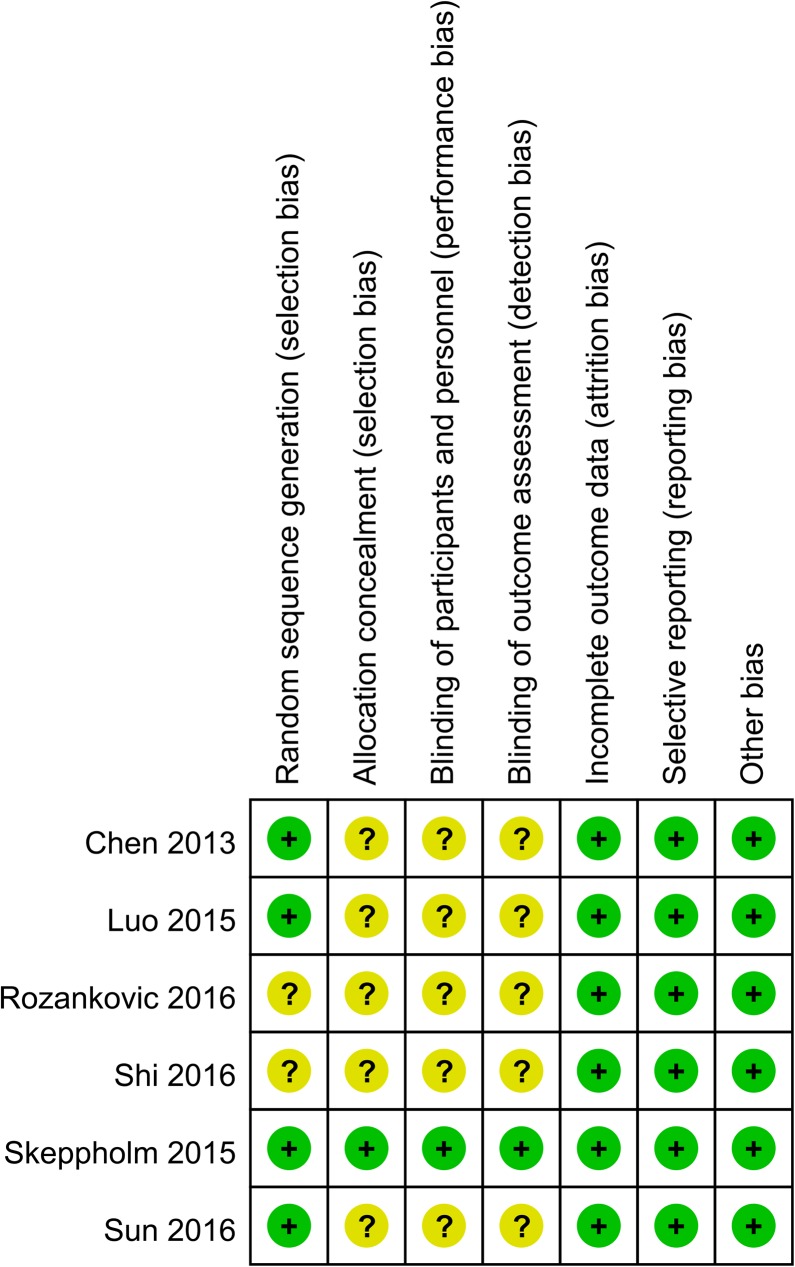
Risk of bias assessment of each included study.

The risk of bias for the included studies are presented in Fig 2. Only one study[22] was regarded as low risk of bias. Although all of the six studies were reported as randomized controlled trials, only four studies[13, 15, 19, 22] showed an appropriate randomization, and one study[22] described allocation concealment detailedly. Only one study[22] reported an adequate blinding for both participants and outcomes assessors.

### Quality of evidence assessment

A summary of the quality of the evidence based on the GRADE approach are displayed in S2 Table. The GRADE level of the evidence was low for NDI scores, neck pain scores, arm pain scores, and secondary surgical procedures; and moderate for operation time, blood loss, ROM at the operative level, JOA scores, and adverse events.

### Outcome analysis of surgical parameters

#### Operation time

**Fig 3:**
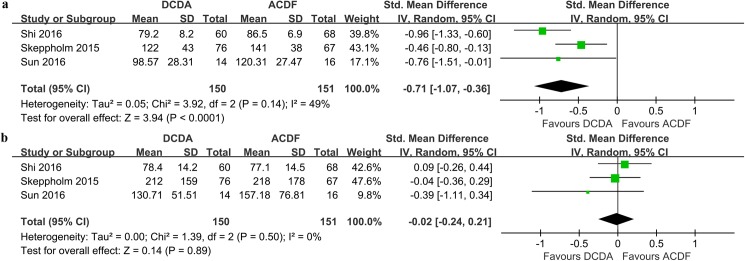
**Forest plots of the included studies comparing operation time (a) and blood loss (b) in patients who underwent DCDA and those who underwent ACDF.** DCDA: discover cervical disc arthroplasty, ACDF: anterior cervical discectomy and fusion.

Three studies[19, 21, 22] with 301 patients (DCDA = 150, ACDF = 151) reported the operation time. The result of our meta-analysis showed that the operation time of DCDA was shorter than that of ACDF, and the difference between the two groups was significant (SMD = -0.71, 95% CI -1.07 ~ -0.36, P < 0.0001; I^2^ = 49%; Fig 3A).

#### Blood loss

We included three studies[19, 21, 22] with 301 patients (DCDA = 150, ACDF = 151) to quantitatively analyze the blood loss between the DCDA group and ACDF group, and no significant difference was observed (SMD = -0.02, 95% CI -0.24 ~ 0.21, P = 0.89; I^2^ = 0%; Fig 3B).

### Outcome analysis of clinical indexes

#### NDI scores

**Fig 4:**
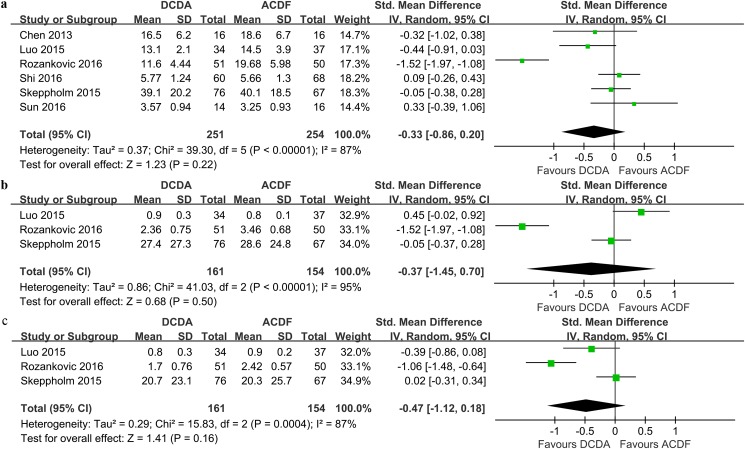
**Forest plots of the included studies comparing NDI scores (a), neck pain scores (b) and arm pain scores (c) in patients who underwent DCDA and those who underwent ACDF.** NDI: neck disability index, DCDA: discover cervical disc arthroplasty, ACDF: anterior cervical discectomy and fusion.

All of the six studies reported the number of patients (n = 505) with the NDI scores. The NDI scores in the DCDA group were comparable to those in the ACDF group (SMD = -0.33, 95% CI -0.86 ~ 0.20, P = 0.22; I^2^ = 87%; Fig 4A).

#### Neck pain scores

Three studies[15, 20, 22] with 315 patients presented the available data for the neck pain scores. No significant difference was found when comparing the neck pain scores between the DCDA and ACDF groups (SMD = -0.37, 95% CI -1.45 ~ 0.70, P = 0.50; I^2^ = 95%; Fig 4B).

#### Arm pain scores

Three studies with 315 patients were pooled to evaluate the arm pain scores of two procedures. Pooled data showed that there was no significant difference between the two groups in arm pain scores (SMD = -0.47, 95% CI -1.12 ~ 0.18, P = 0.16; I^2^ = 87%; Fig 4C).

#### ROM at the operative level

**Fig 5:**
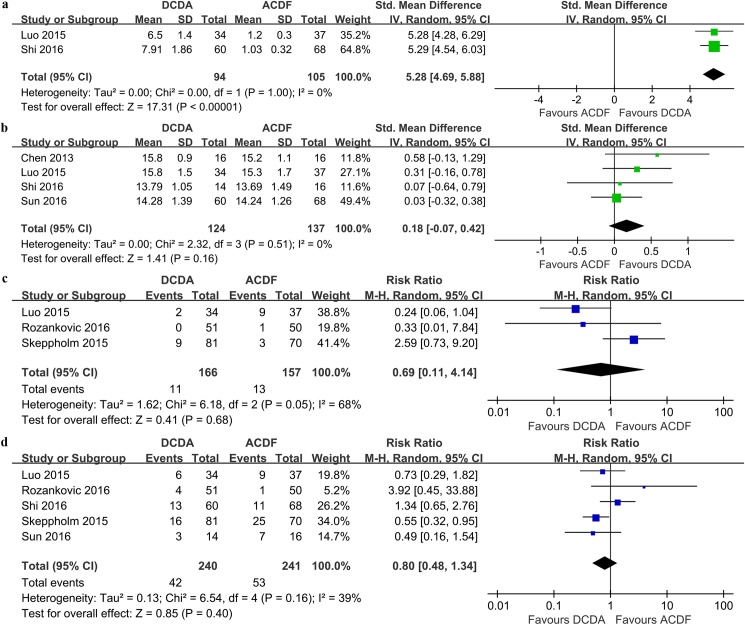
**Forest plots of the included studies comparing ROM at the operative level (a), JOA scores (b), secondary surgical procedures (c) and adverse events (d) in patients who underwent DCDA and those who underwent ACDF.** ROM: range of motion, JOA: Japanese orthopaedic association, DCDA: discover cervical disc arthroplasty, ACDF: anterior cervical discectomy and fusion.

Two studies, including 199 patients, have data available for quantitative analysis of ROM at the operative level. The DCDA group was associated with a significant increase in ROM at the operative level compared to the ACDF group (SMD = 5.28, 95% CI 4.69 ~ 5.88, P < 0.00001; I^2^ = 0%; Fig 5A).

#### JOA scores

Four studies (n = 261) contributed to the analysis of the JOA scores. No significant difference was observed in terms of the JOA scores between the two groups (SMD = 0.18, 95% CI -0.07 ~ 0.42, P = 0.16, I^2^ = 0%; Fig 5B).

#### Secondary surgical procedures

According to the FDA-regulated investigational device exemption (IDE) trial protocol, secondary surgical procedures were defined as revisions, removals, supplemental fixations, or reoperations[10]. Three studies with 323 patients reported the results regarding secondary surgical procedures. And there was no significant difference between the DCDA and ACDF groups (RR = 0.69, 95% CI 0.11 ~ 4.14, P = 0.68; I^2^ = 68%; Fig 5C).

#### Adverse events

All of the six studies provided data for adverse events. We observed similar rates of adverse events when comparing the DCDA group with the ACDF group (RR = 0.80, 95% CI 0.48 ~ 1.34, P = 0.40; I^2^ = 39%; Fig 5D).

### Subgroup analyses, sensitivity analyses, meta-regression analyses and publication bias

Subgroup analyses based on duration of follow-up (short-term (1–3 years) or mid-term (4–5 years)) and target level (single-level or two-level or mixed-level) did not demonstrated significant differences (see S3 Table). The risk increase of adverse events was higher in studies with mixed-level.

Generally speaking, the sensitivity analyses did not alter the results except to use a fixed-effect model for the NDI scores, neck pain scores and arm pain scores, and exclude the largest and most weighted trials for the arm pain scores and **s**econdary surgical procedures (see S4 Table).

Meta-regression analyses demonstrated no effect of female ratio in improving NDI scores and reducing adverse events and no effect of mean age in decreasing adverse events; however, the mean age notably influenced the NDI scores, i.e., trials with higher age showed higher NDI scores (see S1–S4 Figs).

As the number of the studies was less than ten, the publication bias could not be tested.

## Discussion

By reviewing the previous literature, some pervious meta-analyses comparing the effectiveness and safety between CDA and ACDF were found. Lin et al[5] demonstrated that CDA was superior to ACDF for treating SCDD, and Zou et al[9] also indicated that CDA was comparable or superior to ACDF at two contiguous levels cervical disc degenerative diseases. But little was found regarding the comparison of the efficacy and safety between DCDA and ACDF. The recent studies[8, 23–25] demonstrated that different prostheses for CDA might have an influence on the comparing outcomes between CDA and ACDF. Furthermore, various prostheses are available for CDA, and each of them has their own characters which may result in different clinical outcomes. Hence, we performed this meta-analysis to fill the gap and explore whether DCDA was superior to ACDF.

By pooling the most recent randomized controlled trials, we found that the operation time in the DCDA group was shorter than that in the ACDF group. The earlier meta-analyses[26, 27] also compared the operation time between CDA and ACDF and found that CDA was associated with longer operation time. This outcome is different from the present finding. Actually, Murrey et al[28] have declared that as a new surgical technique, the operation for arthroplasty may increase the difficulty of operation, which may result in the increase of operation time. But as time goes on, the operation for arthroplasty became more and more maturely. Meanwhile, the surgeons were also more and more familiar with this procedure, thus the additional use of fluoroscopy decreased during operation, which may contribute to saving the operation time. Moreover, the operation time may be determined by many factors, such as patients’ condition, the actual condition during the operation, the extent of surgeons’ skill and so on[9].

Many previous studies[10, 26, 29] have reported that compared with ACDF, CDA presented lower NDI scores. Zhu et al[30] also found NDI scores of BCDA were statistically lower than those of ACDF. However, this meta-analysis demonstrated that no significant difference was observed between DCDA and ACDF. And only one study with 4-year follow-up was included in mid-term follow-up. Additionally, the sensitivity analyses showed that with a fixed-effect model used for the NDI scores, patients treated with DCDA acquired lower NDI scores in overall-term follow-up. Hence, the result regarding the NDI scores in this meta-analysis was not stable and more studies of high quality with longer follow-up should be performed to verify this finding further. In the present study, the DCDA group acquired similar neck pain, arm pain and JOA scores compared with the ACDF group. These findings were consistent with that regarding similar NDI scores between DCDA and ACDF. Some previous meta-analyses[9, 27] also agreed with these findings. However, Kan et al[24] used traditional and Bayesian meta-analysis and found that CDA was superior to ACDF in terms of neck and arm pain scores. Because the CDA groups in these meta-analyses contained different types of cervical disc prostheses and no subgroup analyses were conducted to assess the influence of different types of prostheses, the findings of the present study is more reliable to compare DCDA and ACDF.

Adjacent segment degeneration was one of the major concerns for patients treated with ACDF. Reginald et al[31] reported that the ROM at the operative level would reduce after ACDF, which may be compensated by increasing the ROM of adjacent level. And compared with ACDF, CDA has an advantage in theoretical biomechanical of motion preservation and stress reduction at adjacent levels[32–35]. Yin et al[36] also concluded that the segment motion at operation level may be retained after CDA, and no significant difference was observed regarding the segment motion at the adjacent level between the CDA group and ACDF group. This concept was confirmed by our meta-analysis. However, Nunley et al[37] found that the risk of adjacent segment degeneration in the CDA group was comparable with the ACDF group. And a biomechanical cadaver study[38] also indicated that all segments were independent with each other. So further studies should be performed to explore the biomechanical and kinematic change at adjacent levels in future.

The DCDA group showed similar adverse events compared with the ACDF group, no matter at short-term or mid-term follow up or for single-level or two-level target level. Zhu et al[30] previously found fewer adverse events began to manifest at the mid-term follow up in the Bryan CDA group. This finding was different from the present finding. Maybe only one trial with mid-term follow up included in the present study contributes to the difference. Heterotopic ossification after implantation of CDA was recongnized as a common complication, which can lead to the loss of ROM of the surgical segment. However, the DCDA group manifested better ROM at the operative level (P < 0.00001) compared with the ACDF group in the present study. Thus heterotopic ossification may do not affect the motion of the prosthesis. Yi et al[39] compared the incidence of heterotopic ossification after three different types of cervical disc prostheses and observed that the heterotopic ossification occurrence rates for the three prostheses showed as follows: 21.0% in the Bryan disc group; 52.5% in the Mobi-C group; and 71.4% in the ProDisc C group. Qi et al[40] found the incidence of heterotopic ossification was 24.8% in the Discover disc group, which was analogous to those in other different prostheses. Dysphagia was reported as the most frequent complication of ACDF, which comprised almost 40% of total adverse events[41]. Dysphagia is significantly lower in the DCDA group than the ACDF group, because DCDA requires less esophageal retraction and subsequently decreases the intra-esophageal pressure[7].

The strengths of our study are presented as follows. First, to our knowledge, this study was the first meta-analysis to evaluate the efficacy and safety between DCDA and ACDF. Second, we made a rigorous search strategy, and all of the randomized controlled trials meeting our criteria were included. Third, we adhered to the Preferred Reporting Items for Systematic Reviews and Meta-Analyses (PRISMA) standards to perform and report our meta-analysis, which made this study more rigorous and credible.

However, there were also some limitations which must be declared in our study. First, given that the number of trials available on this topic was relatively small, the estimates and the statistical power can’t be ensured. Additionally, this study consisted of six trials, but half of them had a modest sample size (n < 100). Compared with large sample size trials, trials with small sample size trended to overrate the treatment effectiveness, which may restrict the power of the judgments. Second, in terms of mid-term follow-up, only one study was included. So we couldn’t pool data and estimate the pooled effect. Besides, in terms of long-term follow-up, no studies were retrieved and included, and the outcomes of long-term follow-up couldn’t be assessed. Third, only one study was regarded as low risk of bias and other studies were at unclear risk of bias, which may influence the outcomes, so more studies with high quality were needed in future.

## Conclusions

In conclusion, compared with ACDF, DCDA demonstrated shorter operation time and better ROM at the operative level. However, no significant differences were observed in blood loss, NDI scores, neck and arm pain scores, JOA scores, secondary surgery procedures and adverse events. Additionally, more studies of high quality with mid- to long-term follow-up are required in future.

## Supporting information

S1 PRISMA Checklist (DOC)

S1 Fig Meta-regression analysis of influence of female ratio on NDI scores of Discover cervical disc arthroplasty. The circles represent each study. The size of the circle represents the power of the study. The solid line indicates the weighted regression line. NDI: neck disability index.
(TIF)

S2 Fig Meta-regression analysis of influence of female ratio on adverse events of Discover cervical disc arthroplasty. The circles represent each study. The size of the circle represents the power of the study. The solid line indicates the weighted regression line.
(TIF)

S3 Fig Meta-regression analysis of influence of mean age on adverse events of Discover cervical disc arthroplasty. The circles represent each study. The size of the circle represents the power of the study. The solid line indicates the weighted regression line.
(TIF)

S4 Fig Meta-regression analysis of influence of mean age on NDI scores of Discover cervical disc arthroplasty. The circles represent each study. The size of the circle represents the power of the study. The solid line indicates the weighted regression line. NDI: neck disability index.
(TIF)

S1 Table Search strategies. (DOCX)

S2 Table GRADE evidence profile. (DOCX)

S3 Table Subgroup analyses. (DOCX)

S4 Table Sensitivity analyses. (DOCX)
