# Supplementary figures and images for: Discover cervical disc arthroplasty versus anterior cervical discectomy and fusion in symptomatic cervical disc diseases: A meta-analysis
Source: PLoS One. 2017 Mar 30;12(3):e0174822. doi: 10.1371/journal.pone.0174822 (PMC5373642; doi:10.1371/journal.pone.0174822)

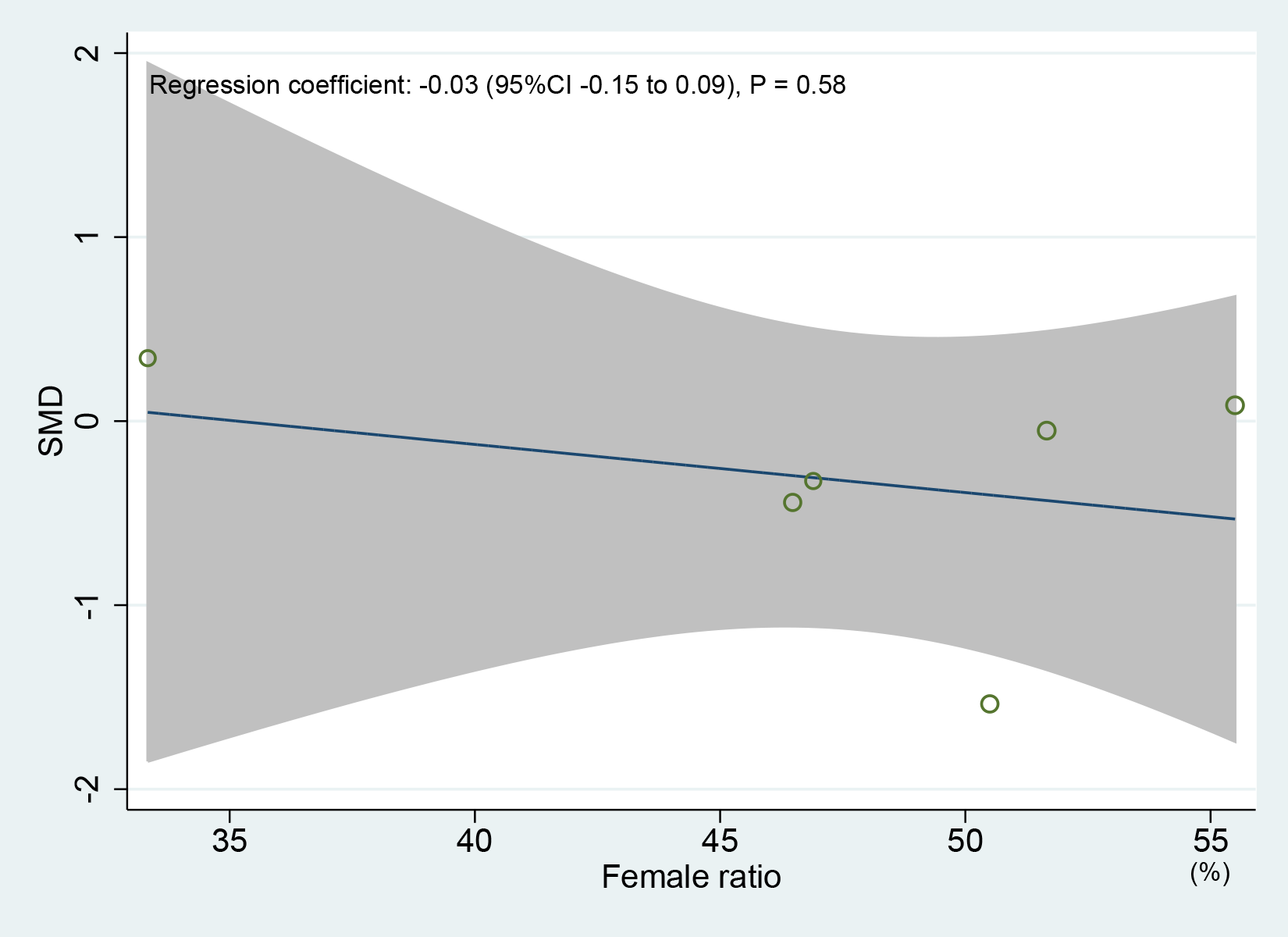

Supplement: S1 Fig — The circles represent each study. The size of the circle represents the power of the study. The solid line indicates the weighted regression line. NDI: neck disability index. (TIF) [file pone.0174822.s002.tif]

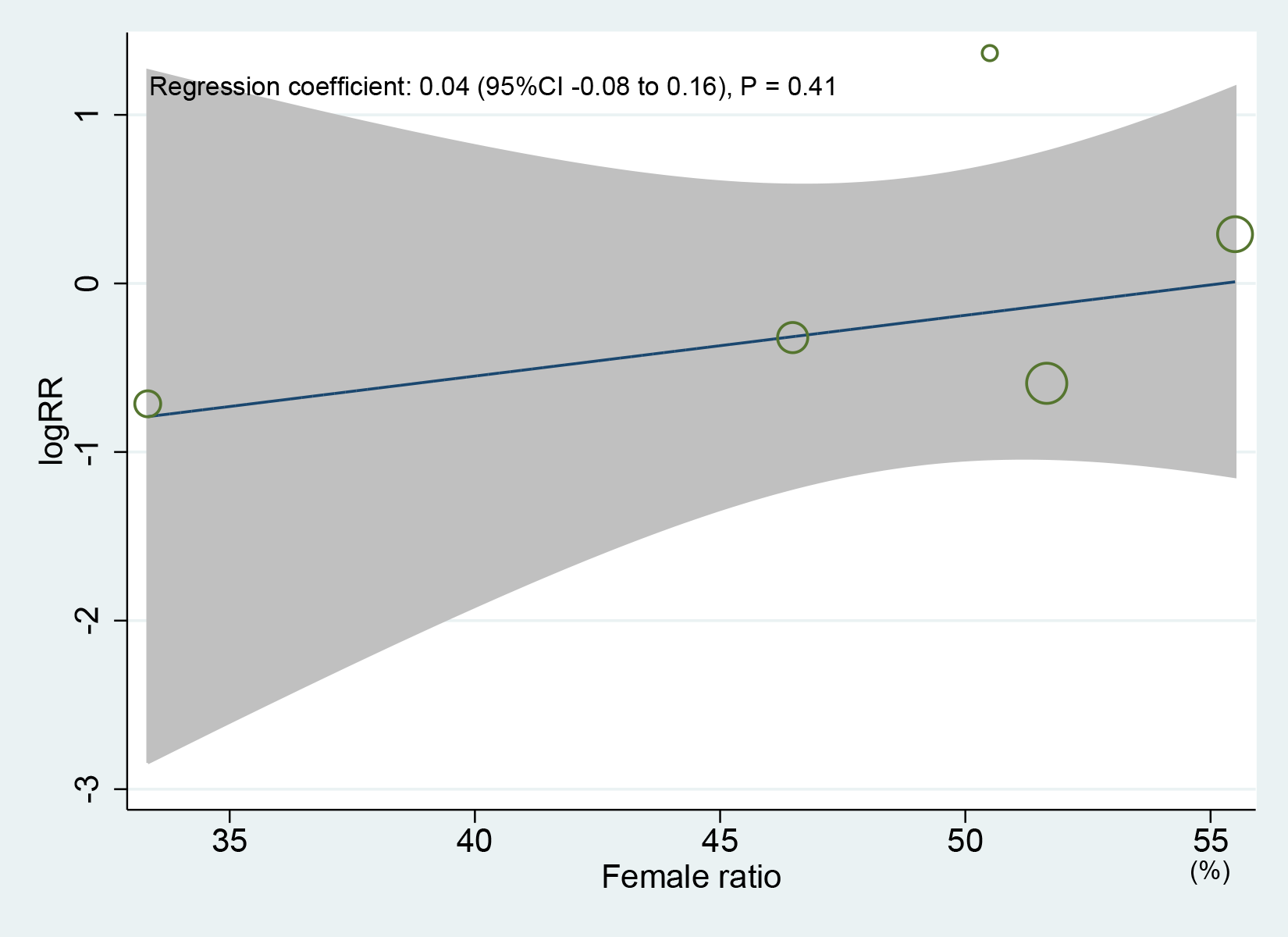

Supplement: S2 Fig — The circles represent each study. The size of the circle represents the power of the study. The solid line indicates the weighted regression line. (TIF) [file pone.0174822.s003.tif]

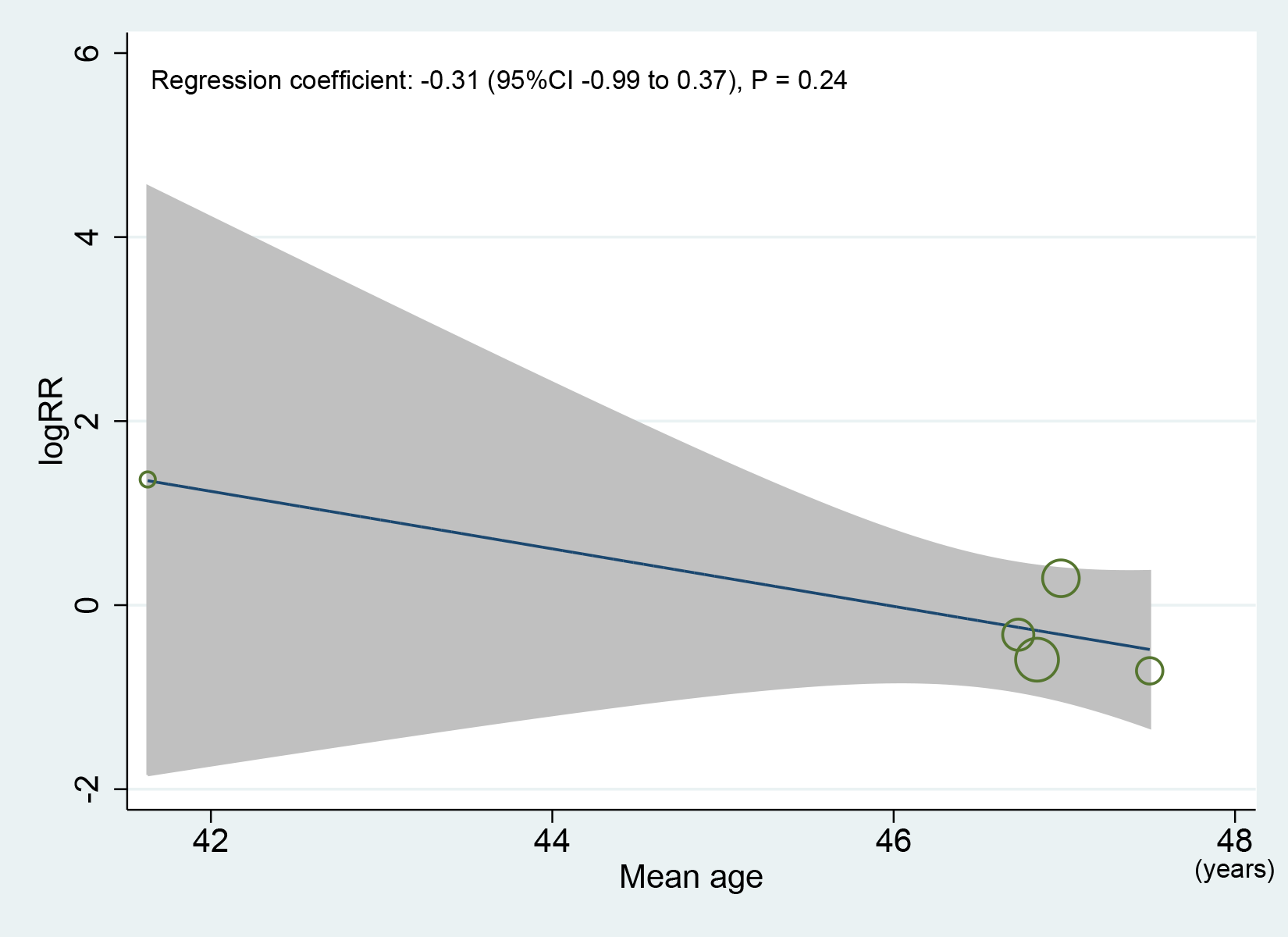

Supplement: S3 Fig — The circles represent each study. The size of the circle represents the power of the study. The solid line indicates the weighted regression line. (TIF) [file pone.0174822.s004.tif]

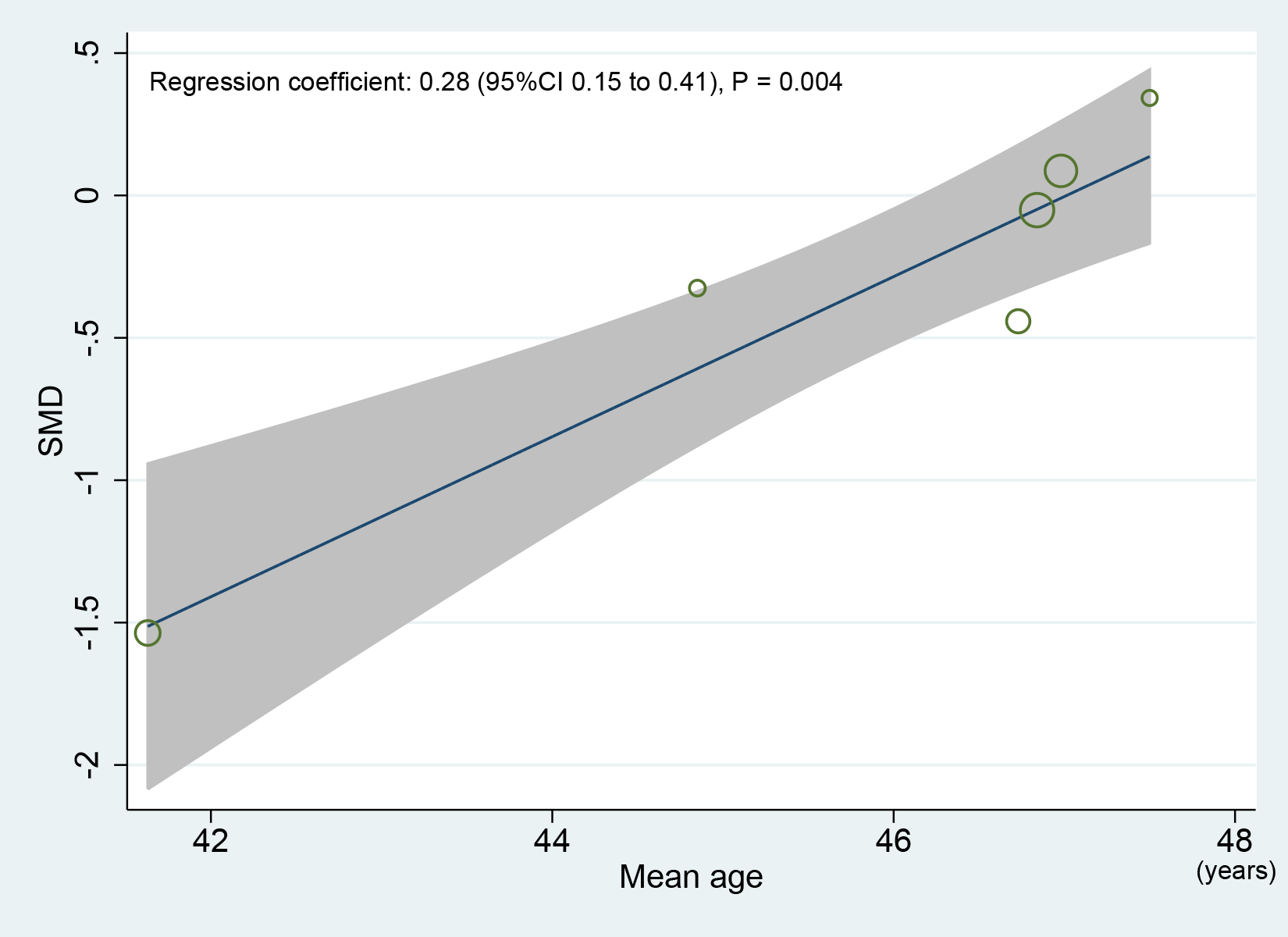

Supplement: S4 Fig — The circles represent each study. The size of the circle represents the power of the study. The solid line indicates the weighted regression line. NDI: neck disability index. (TIF) [file pone.0174822.s005.tif]
