# Supplementary material for: Discover cervical disc arthroplasty versus anterior cervical discectomy and fusion in symptomatic cervical disc diseases: A meta-analysis
Source: PLoS One. 2017 Mar 30;12(3):e0174822. doi: 10.1371/journal.pone.0174822 (PMC5373642; doi:10.1371/journal.pone.0174822)
Supplement: S1 Table — (DOCX) [file pone.0174822.s006.docx]

**S1 Table. Search strategies.**

**PubMed**

| **Search** | **Query** |
| --- | --- |
| #1 | "Randomized Controlled Trial" [Publication Type] |
| #2 | "Controlled Clinical Trial" [Publication Type] |
| #3 | ((((randomized[Title/Abstract]) OR placebo[Title/Abstract]) OR randomly[Title/Abstract]) OR trial[Title/Abstract]) OR group[Title/Abstract] |
| #4 | #1 OR #2 OR #3 |
| #5 | "Total Disc Replacement"[Mesh] |
| #6 | (disk*[Title/Abstract]) OR disc*[Title/Abstract] |
| #7 | (replac*[Title/Abstract]) OR prosthes*[Title/Abstract] |
| #8 | #6 AND #7 |
| #9 | (cerv*[Title/Abstract]) AND arthroplast*[Title/Abstract] |
| #10 | (spin*[Title/Abstract]) AND arthroplast*[Title/Abstract] |
| #11 | #6 AND artificial[Title/Abstract] |
| #12 | ((((((total disc arthroplast*[Title/Abstract]) OR total disc replacement*[Title/Abstract]) OR TDA[Title/Abstract]) OR total cervical disc arthroplast*[Title/Abstract]) OR TCDA[Title/Abstract]) OR prosthes*[Title/Abstract]) OR dynamic device*[Title/Abstract] |
| #13 | #5 OR #8 OR #9 OR #10 OR #11 OR #12 |
| #14 | "Spinal Fusion"[Mesh] |
| #15 | (spin*[Title/Abstract]) AND fusion[Title/Abstract] |
| #16 | (cerv*[Title/Abstract]) AND fusion[Title/Abstract] |
| #17 | "Diskectomy"[Mesh] |
| #18 | (cerv*[Title/Abstract]) AND discectomy[Title/Abstract] |
| #19 | (cerv*[Title/Abstract]) AND diskectomy[Title/Abstract] |
| #20 | (((anterior cervical discectomy[Title/Abstract] AND fusion[Title/Abstract])) OR ACDF[Title/Abstract]) OR cervical spine arthrodesis[Title/Abstract] |
| #21 | #14 OR #15 OR #16 |
| #22 | #17 OR #18 OR #19 |
| #23 | #21 AND #22 |
| #24 | #20 OR #23 |
| #25 | Discover[Title/Abstract] |
| #26 | #4 AND #13 AND #24 AND #25 |
