# Supplementary material for: Discover cervical disc arthroplasty versus anterior cervical discectomy and fusion in symptomatic cervical disc diseases: A meta-analysis
Source: PLoS One. 2017 Mar 30;12(3):e0174822. doi: 10.1371/journal.pone.0174822 (PMC5373642; doi:10.1371/journal.pone.0174822)
Supplement: S2 Table — (DOCX) [file pone.0174822.s007.docx]

**S2 Table. GRADE evidence profile.**

| **Quality assessment** | | | | | | | **No of patients** | | **Effect** | | **Quality*** | **Importance** |
| --- | --- | --- | --- | --- | --- | --- | --- | --- | --- | --- | --- | --- |
|  |  |  |  |  |  |  |  |  |  |  |  |  |
| **No of studies** | **Design** | **Risk of bias** | **Inconsistency** | **Indirectness** | **Imprecision** | **Other considerations** | **DCDA** | **ACDF** | **Relative (95% CI)** | **Absolute** |  |  |
| **Operation time (follow-up 24-32.4 months)** | | | | | | | | | | | | |
| 3 | randomised trials | serious^1^ | no serious inconsistency | no serious indirectness | no serious imprecision | none | 150 | 151 | - | SMD 0.71 lower (1.07 to 0.36 lower) | ⊕⊕⊕O MODERATE | IMPORTANT |
| **Blood loss (follow-up 24-32.4 months)** | | | | | | | | | | | | |
| 3 | randomised trials | serious^1^ | no serious inconsistency | no serious indirectness | no serious imprecision | none | 150 | 151 | - | SMD 0.02 lower (0.24 lower to 0.21 higher) | ⊕⊕⊕O MODERATE | IMPORTANT |
| **NDI scores (follow-up 24-48 months)** | | | | | | | | | | | | |
| 6 | randomised trials | serious^1^ | serious^2^ | no serious indirectness | no serious imprecision | none | 251 | 254 | - | SMD 0.33 lower (0.86 lower to 0.2 higher) | ⊕⊕OO LOW | CRITICAL |
| **Neck pain scores (follow-up 24-48 months)** | | | | | | | | | | | | |
| 3 | randomised trials | serious^1^ | serious^3^ | no serious indirectness | no serious imprecision | none | 161 | 154 | - | SMD 0.37 lower (1.45 lower to 0.7 higher) | ⊕⊕OO LOW | CRITICAL |
| **Arm pain scores (follow-up 24-48 months)** | | | | | | | | | | | | |
| 3 | randomised trials | serious^1^ | serious^4^ | no serious indirectness | no serious imprecision | none | 161 | 154 | - | SMD 0.47 lower (1.12 lower to 0.18 higher) | ⊕⊕OO LOW | CRITICAL |
| **ROM (follow-up 24-48 months)** | | | | | | | | | | | | |
| 2 | randomised trials | serious^5^ | no serious inconsistency | no serious indirectness | no serious imprecision | none | 94 | 105 | - | SMD 5.28 higher (4.69 to 5.88 higher) | ⊕⊕⊕O MODERATE | CRITICAL |
| **JOA scores (follow-up 24-48 months)** | | | | | | | | | | | | |
| 4 | randomised trials | serious^5^ | no serious inconsistency | no serious indirectness | no serious imprecision | none | 124 | 137 | - | SMD 0.18 higher (0.07 lower to 0.42 higher) | ⊕⊕⊕O MODERATE | CRITICAL |
| **Secondary surgical procedures (follow-up 24-48 months)** | | | | | | | | | | | | |
| 3 | randomised trials | serious^1^ | serious^6^ | no serious indirectness | no serious imprecision | none | 11/166  (6.6%) | 13/157  (8.3%) | RR 0.69 (0.11 to 4.14) | 26 fewer per 1000 (from 74 fewer to 260 more) | ⊕⊕OO LOW | CRITICAL |
|  |  |  |  |  |  |  |  | 4.3% |  | 13 fewer per 1000 (from 38 fewer to 135 more) |  |  |
| **Adverse events (follow-up 24-48 months)** | | | | | | | | | | | | |
| 5 | randomised trials | serious^1^ | no serious inconsistency | no serious indirectness | no serious imprecision | none | 42/240  (17.5%) | 53/241  (22%) | RR 0.8 (0.48 to 1.34) | 44 fewer per 1000 (from 114 fewer to 75 more) | ⊕⊕⊕O MODERATE | CRITICAL |
|  |  |  |  |  |  |  |  | 24.3% |  | 49 fewer per 1000 (from 126 fewer to 83 more) |  |  |

DCDA: Discover cervical disc arthroplasty; ACDF: anterior cervical discectomy and fusion; NDI: neck disability index; ROM: range of motion; JOA: Japanese orthopaedic association.

^1^ Almost all the trials were judged to be at unclear risk of bias.
^2^ Significant heterogeneity (I^2^ = 87%) was found.
^3^ Significant heterogeneity (I^2^ = 95%) was found.
^4^ Significant heterogeneity (I^2^ = 87%) was found.

^5^ All the trials were judged to be at unclear risk of bias.
^6^ Significant heterogeneity (I^2^ = 68%) was found.

*GRADE Working Group grades of evidence: high quality = further research is very unlikely to change our confidence in the estimate of effect; moderate quality = further research is likely to have an important impact on our confidence in the estimate of effect and may change the estimate; low quality = further research is very likely to have an important impact on our confidence in the estimate of effect and is likely to change the estimate; very low quality = we are very uncertain about the estimate.
