# Supplementary material for: Discover cervical disc arthroplasty versus anterior cervical discectomy and fusion in symptomatic cervical disc diseases: A meta-analysis
Source: PLoS One. 2017 Mar 30;12(3):e0174822. doi: 10.1371/journal.pone.0174822 (PMC5373642; doi:10.1371/journal.pone.0174822)
Supplement: S3 Table — (DOCX) [file pone.0174822.s008.docx]

**S2 Table. Subgroup analyses.**

| **Subgroup** | **Sample size** | **Measures of effects size and precision** | | | **Heterogeneity** |
| --- | --- | --- | --- | --- | --- |
|  |  | **Point** **estimate** | **95% confidence interval** | **P value** | **I^2^** |
| **NDI scores** |  |  |  |  |  |
| Total | 505 | -0.33 | -0.86 to 0.20 | 0.22 | 87% |
| Duration of follow-up |  |  |  |  |  |
| Short-term | 434 | -0.31 | -0.95 to 0.34 | 0.35 | 90% |
| Mid-term | 71 | -0.44 | -0.91 to 0.03 | 0.07 | Not applicable |
| Target level |  |  |  |  |  |
| Single-level | 332 | -0.55 | -1.31 to 0.21 | 0.16 | 91% |
| Two-level | 30 | 0.33 | -0.39 to 1.06 | 0.37 | Not applicable |
| Mixed-level | 143 | -0.05 | -0.38 to 0.28 | 0.76 | Not applicable |
| **Neck pain scores** |  |  |  |  |  |
| Total | 315 | -0.37 | -1.45 to 0.70 | 0.50 | 95% |
| Duration of follow-up |  |  |  |  |  |
| Short-term | 244 | -0.78 | -2.23 to 0.67 | 0.29 | 96% |
| Mid-term | 71 | 0.45 | -0.02 to 0.92 | 0.06 | Not applicable |
| **Arm pain scores** |  |  |  |  |  |
| Total | 315 | -0.47 | -1.12 to 0.18 | 0.16 | 87% |
| Duration of follow-up |  |  |  |  |  |
| Short-term | 244 | -0.51 | -1.57 to 0.54 | 0.34 | 94% |
| Mid-term | 71 | -0.39 | -0.86 to 0.08 | 0.10 | Not applicable |
| **ROM** |  |  |  |  |  |
| Total | 199 | 5.28 | 4.69 to 5.88 | < 0.00001 | 0% |
| Duration of follow-up |  |  |  |  |  |
| Short-term | 128 | 5.29 | 4.54 to 6.03 | < 0.00001 | Not applicable |
| Mid-term | 71 | 5.28 | 4.28 to 6.29 | < 0.00001 | Not applicable |
| **JOA scores** |  |  |  |  |  |
| Total | 261 | 0.18 | -0.07 to 0.42 | 0.16 | 0% |
| Duration of follow-up |  |  |  |  |  |
| Short-term | 190 | 0.13 | -0.16 to 0.41 | 0.38 | 0% |
| Mid-term | 71 | 0.31 | -0.16 to 0.78 | 0.20 | Not applicable |
| Target level |  |  |  |  |  |
| Single-level | 133 | 0.32 | -0.02 to 0.66 | 0.07 | 0% |
| Two-level | 128 | 0.03 | -0.32 to 0.38 | 0.87 | Not applicable |
| **Secondary surgical procedures** |  |  |  |  |  |
| Total | 323 | 0.69 | 0.11 to 4.14 | 0.68 | 68% |
| Duration of follow-up |  |  |  |  |  |
| Short-term | 252 | 1.57 | 0.27 to 8.97 | 0.61 | 29% |
| Mid-term | 71 | 0.24 | 0.06 to 1.04 | 0.06 | Not applicable |
| **Adverse events** |  |  |  |  |  |
| Total | 481 | 0.80 | 0.48 to 1.34 | 0.40 | 39% |
| Duration of follow-up |  |  |  |  |  |
| Short-term | 410 | 0.85 | 0.43 to 1.68 | 0.63 | 54% |
| Mid-term | 71 | 0.73 | 0.29 to 1.82 | 0.50 | Not applicable |
| Target level |  |  |  |  |  |
| Single-level | 300 | 1.16 | 0.62 to 2.20 | 0.64 | 17% |
| Two-level | 30 | 0.49 | 0.16 to 1.54 | 0.22 | Not applicable |
| Mixed-level | 151 | 0.55 | 0.32 to 0.95 | 0.03 | Not applicable |

DCDA: Discover cervical disc arthroplasty; ACDF: anterior cervical discectomy and fusion; NDI: neck disability index; ROM: range of motion; JOA: Japanese orthopaedic association.
