# Supplementary material for: Discover cervical disc arthroplasty versus anterior cervical discectomy and fusion in symptomatic cervical disc diseases: A meta-analysis
Source: PLoS One. 2017 Mar 30;12(3):e0174822. doi: 10.1371/journal.pone.0174822 (PMC5373642; doi:10.1371/journal.pone.0174822)
Supplement: S4 Table — (DOCX) [file pone.0174822.s009.docx]

**S3 Table. Sensitivity analyses.**

| **Sensitivity analysis** | **Measures of effects size and precision** | | | **Heterogeneity** |
| --- | --- | --- | --- | --- |
|  | **Point** **estimate** | **95%CI** | **P value** | **I^2^** |
| **NDI scores** |  |  |  |  |
| All studies (random-effect model with SMD) | -0.33 | -0.86 to 0.20 | 0.22 | 87% |
| All studies (fixed-effect model with SMD) | -0.30 | -0.48 to -0.12 | 0.0009 | 87% |
| Excluding the largest trial | -0.39 | -1.06 to 0.29 | 0.26 | 89% |
| Excluding the most weighted trial | -0.39 | -1.06 to 0.29 | 0.26 | 89% |
| **Neck pain scores** |  |  |  |  |
| All studies (random-effect model with SMD) | -0.37 | -1.45 to 0.70 | 0.50 | 95% |
| All studies (fixed-effect model with SMD) | -0.32 | -0.55 to -0.09 | 0.006 | 95% |
| Excluding the largest trial | -0.54 | -2.47 to 1.40 | 0.59 | 97% |
| Excluding the most weighted trial | -0.54 | -2.47 to 1.40 | 0.59 | 97% |
| **Arm pain scores** |  |  |  |  |
| All studies (random-effect model with SMD) | -0.47 | -1.12 to 0.18 | 0.16 | 87% |
| All studies (fixed-effect model with SMD) | -0.39 | -0.62 to -0.17 | 0.0006 | 87% |
| Excluding the largest trial | -0.74 | -1.39 to -0.08 | 0.03 | 77% |
| Excluding the most weighted trial | -0.74 | -1.39 to -0.08 | 0.03 | 77% |
| **ROM** |  |  |  |  |
| All studies (random-effect model with SMD) | 5.28 | 4.69 to 5.88 | < 0.00001 | 0% |
| All studies (fixed-effect model with SMD) | 5.28 | 4.69 to 5.88 | < 0.00001 | 0% |
| Excluding the largest trial | 5.28 | 4.28 to 6.29 | < 0.00001 | Not applicable |
| Excluding the most weighted trial | 5.28 | 4.28 to 6.29 | < 0.00001 | Not applicable |
| **JOA scores** |  |  |  |  |
| All studies (random-effect model with SMD) | 0.18 | -0.07 to 0.42 | 0.16 | 0% |
| All studies (fixed-effect model with SMD) | 0.18 | -0.07 to 0.42 | 0.16 | 0% |
| Excluding the largest trial | 0.32 | -0.02 to 0.66 | 0.07 | 0% |
| Excluding the most weighted trial | 0.32 | -0.02 to 0.66 | 0.07 | 0% |
| **Secondary surgical procedures** |  |  |  |  |
| All studies (random-effect model with RR) | 0.69 | 0.11 to 4.14 | 0.68 | 68% |
| All studies (fixed-effect model with RR) | 0.82 | 0.37 to 1.79 | 0.61 | 68% |
| Excluding the largest trial | 0.25 | 0.07 to 0.96 | 0.04 | 0% |
| Excluding the most weighted trial | 0.25 | 0.07 to 0.96 | 0.04 | 0% |
| **Adverse events** |  |  |  |  |
| All studies (random-effect model with RR) | 0.80 | 0.48 to 1.34 | 0.40 | 39% |
| All studies (fixed-effect model with RR) | 0.79 | 0.55 to 1.13 | 0.19 | 39% |
| Excluding the largest trial | 0.97 | 0.52 to 1.80 | 0.92 | 28% |
| Excluding the most weighted trial | 0.97 | 0.52 to 1.80 | 0.92 | 28% |

CI: confidence interval; SMD: standardized mean difference; RR: relative risk; NDI: neck disability index; ROM: range of motion; JOA: Japanese orthopaedic association.
